# Adsorption properties of Pb(II) and Cd(II) in acid mine drainage by oyster shell loaded lignite composite in different morphologies

**DOI:** 10.1038/s41598-024-62506-0

**Published:** 2024-05-21

**Authors:** Wenbo An, Yifan Liu, He Chen, Xueying Sun, Qiqi Wang, Xuechun Hu, Junzhen Di

**Affiliations:** 1 Ordos Institute of Liaoning Technical University, Ordos, 017000 China; 2 School of Civil Engineering, Liaoning Technical University, 88 Yulong Road, Xihe District, Fuxin City, 123000 Liaoning Province China; 3 School of Mechanics and Engineering, Liaoning Technical University, Fuxin, 123000 China; 4 Shanghai Chemical Industrial Zone Sino-French Water Development Co., LTD, Shanghai, 200000 China

**Keywords:** Acid mine drainage, Lignite, Oyster shell, Adsorbent morphology, Adsorption, Heavy metal, Environmental impact, Pollution remediation

## Abstract

A new idea to alleviate environmental pollution is the development of low-cost adsorbents using natural minerals and fishery wastes to treat high concentrations of heavy metal pollutants in acid mine drainage (AMD). Adsorbent morphology, adsorptive and regenerative capacity, and application potential are limiting factors for their large-scale use. Oyster shells capable of releasing alkalinity were loaded on the surface of lignite to develop two composite adsorbents with different morphologies (powdery and globular) for the treatment of AMD containing Pb(II) and Cd(II). The results show that the ability of the adsorbent to treat AMD is closely related to its morphologies. The pseudo-second-order kinetic model and the Langmuir model are suitable to describe the adsorption process of OS-M(P), and the maximum adsorption saturation capacities of Pb(II) and Cd(II) are 332.6219 mg/g and 318.9854 mg/g, respectively. The pseudo-second-order kinetic model and the Freundlich model are suitable to describe the adsorption process of OS-M(G). A synergistic result of electrostatic adsorption, neutralization precipitation, ion exchange and complex reaction is achieved in the removal of Pb(II) and Cd(II) by two morphologies of adsorbents. The regeneration times (5 times) and recovery rate (75.75%) of OS-M(G) are higher than those of OS-M(P) (3 times) and recovery rate (20%). The ability of OS-M(G) to treat actual AMD wastewater is still better than that of OS-M(P). OS-M(G) can be used as a promising environmentally friendly adsorbent for the long-term remediation of AMD. This study provides a comprehensive picture of resource management and reuse opportunities for natural mineral and fishery wastes.

## Introduction

During mining, flotation and tailings disposal, sulfide minerals such as tailings and waste rock (primarily pyrite and FeS_2_) are exposed to air and water. They form AMD through physical and chemical reactions caused by rain and microorganisms^1,2^. AMD has been recognized as a major source of soil and water pollution in mining areas around the world due to its characteristics of high acidity, high sulfate concentration and heavy metals^3–7^. If it is directly discharged into groundwater or surface water without treatment, it will cause a number of serious environmental pollution problems and even destroy the ecological balance^8,9^. It is worth mentioning that Pb and Cd have a high level of toxicity and will cause irreversible damage to the human body^10^. Cd in particular is readily absorbed by plants in acidic environments, and acute symptoms of poisoning such as vomiting occur quickly after ingestion. Pb travels with the blood to the brain and causes damage to the cerebellum and cerebral cortex, resulting in cumulative poisoning. In addition, Cd and Pb are potential carcinogens and are the main elements threatening human health. In the process of developing mineral resources, AMD is a major challenge for the global mining industry^11^. The U.S. EPA considers AMD pollution to be second only to “global warming” and the “hole in the ozone layer” in ecological risk^12^. Therefore, an effective method is urgently needed to solve the problem of AMD pollution, especially to prevent or reduce harm to the environment from Pb and Cd.

Various methods have been developed to remove heavy metal elements from AMD, such as constructed wetland method^13,14^, microbial method^15,16^, neutralization precipitation method^17^, and adsorption method^18–20^. Among these methods, the adsorption method is an effective method for the removal of heavy metal ions because of its advantages, which are easy operation, mild adsorption conditions, good adaptability and high efficiency. However, it has become a trend to find efficient, low-cost and readily available adsorbents (activated sludge^21,22^, industrial waste^23,24^, shell powder^22,25^, zeolite^26^, steel slag^27^, agricultural and forestry waste^28^) to replace the traditional expensive adsorbents. Among many low-cost adsorbents, lignite stands out because it is a natural mineral with low calorific value upon combustion. Lignite has a good application potential in the field of wastewater treatment due to its wide distribution, abundant reserves, high specific surface area, developed pore structure, and abundance of active oxygen-containing groups^29–31^. Bao et al. used natural lignite for the treatment of AMD containing Cu(II) and Zn(II), and found that the saturated adsorption capacities were 55.5 mg/g and 67.84 mg/g, respectively^32^. Obviously, natural lignite as an adsorbent for the repair of AMD has the advantages of economy, easy availability and excellent effect. However, considering the adsorption capacity, adsorption selectivity and neutralizing acidity of natural lignite, it is often modified (loaded with other materials to prepare composite adsorbents). Huang et al. modified the lignite with nitric acid, which can enhance its surface electronegativity, polarity, and hydrophilicity, and increase the adsorption capacity for Pb(II) from 14.45 to 30.68 mg/g^33^. Wang et al. prepared a Ca-CHM and used it for adsorption of Cd(II), the *q*_max_ was 41.84 mg/g^34^. In addition, considering the strong acidity of AMD, the choice of loaded alkaline materials is an effective way.

As a solid marine waste, oyster shells accumulate in large quantities along coastlines. They generate odors, breed insects, and pollute water supplies. These negative factors are not only a waste of resources. They also cause serious ecological damage^35–38^. The surface of oyster shell is porous, rich in CaCO_3_ and CaO, alkaline, and has good potential to neutralize acid and absorb heavy metals^39^. Yen and Li^40^ have found that when the oyster shell is treated at a high temperature, its carbonate components are decomposed into a more alkaline form of CaO, which is accompanied by the release of CO_2_. This is beneficial to improve the alkalinity release capacity and increase the porosity of the oyster shell, but also leads to increased agglomeration of the oyster shell powder. Shi et al. prepared FMBO/OS composites by loading iron-manganese oxide (FMBO) on calcined oyster shell by hydrothermal method, which not only solved the defects of FMBO particles being difficult to separate and easy to agglomerate, but also effectively adsorbed and removed As(II) in water^41^. Therefore, oyster shell can be considered to be combined with other materials with good water treatment potential to solve the limitation of a single adsorbent material to treat pollutants and enhance its water treatment capacity. Liu et al. found that the adsorption capacity of lead from oyster shell pow-peanut shell biochar was 27 mg/g^42^. Gao et al. prepared Ca-biochar from oyster shell waste combined with wood waste for the remediation of AMD containing As(III). The medium-speed ball milling method can avoid particle aggregation and release Ca (89.0 mg/g) and alkalinity, and the removal rate of As(III) is 74.0%^43^. However, both lignite powder and oyster shell powder have the advantages of large specific surface area and good adsorption performance, but powder adsorbent also has the disadvantages of difficult solid–liquid separation, transmission loss and poor permeability due to its small particle size and difficult settling characteristics. Some studies have shown that molding treatment can solve the disadvantages of powder adsorbent, but the adsorption capacity is not as good as that of powder materials^44–46^. Therefore, the morphology, regeneration ability and application potential of the adsorbents are important limitations for the large-scale application of the developed adsorbents.

Current studies have mainly explored the characteristics of single form adsorbents. However, the comparative study of the characteristics of different morphologies of adsorbents has not been involved. Therefore, the development of two low-cost composite adsorbents for the treatment of AMD containing Pb(II) and Cd(II) is the main objective of this study. The natural mineral (lignite) with good adsorption effect for heavy metals as the carrier, load marine solid waste (oyster shell) that can release alkalinity. The two adsorbents with different morphologies are namely oyster shell loaded lignite composite adsorbent in powdery morphology (OS-M(P)) and oyster shell loaded lignite composite adsorbent in globular morphology (OS-M(G)). The adsorption properties of the two adsorbents were investigated and compared through batch experiments, the potential adsorption mechanism was explored, the heavy metal leaching and regeneration capacity of the saturated adsorbents were evaluated, and the treatment capacity for the actual AMD was investigated to evaluate the application potential of the two adsorbents. The novelty of this study is to compare the advantages and disadvantages of the two adsorbents in terms of adsorption properties, regeneration ability and application potential. It can provide a new solution to control AMD pollution and reduce the pressure of environmental management caused by the irrational use of lignite and oyster shells.

## Material and method

### Materials, chemicals, and simulated AMD

The lignite used in this experiment was purchased from Shanxi Fuhong Mineral Products Co., LTD. Before use, it is cleaned several times and dried in a drying oven at 105 °C until it reaches a constant weight, crushed and screened 80 to 100 mesh with a high-speed crusher. The oyster shells used in this experiment were collected from kitchen waste. After cleaning with deionized water several times, they were drying at 105 °C for 48 h, crushed with a high-speed crusher, and sieved 100–200 mesh. The bentonite used in this experiment is calcium bentonite (< 200 mesh) purchased from Shanlinshiyu Mineral Products Co., LTD., Xiangxi, Hunan. Nitric acid was purchased from Shenyang Huadong Reagent Factory (Shenyang, China), lead nitrate, cadmium nitrate and sodium hydroxide were purchased from Liaoning Quanrui Reagent Co., LTD. (Jinzhou, China). The simulated AMD is based on the concentration range of contaminants in mine water from a mining company in Huludao, Liaoning Province, China. The pure grades of Pb(NO_3_)_2_ and Cd(NO_3_)_2_·4H_2_O were used as raw materials to simulate Pb(II) and Cd(II) AMD. The pH was adjusted by adding 0.1M HNO_3_ or 0.1M NaOH solution. The initial concentrations of Pb(II) and Cd(II) were 50 mg/L and 10 mg/L, respectively, and the pH of the solution was 4.0. All experimental water samples are used in the current configuration and are not retained.

### Adsorbent preparation

**Figure 1:**
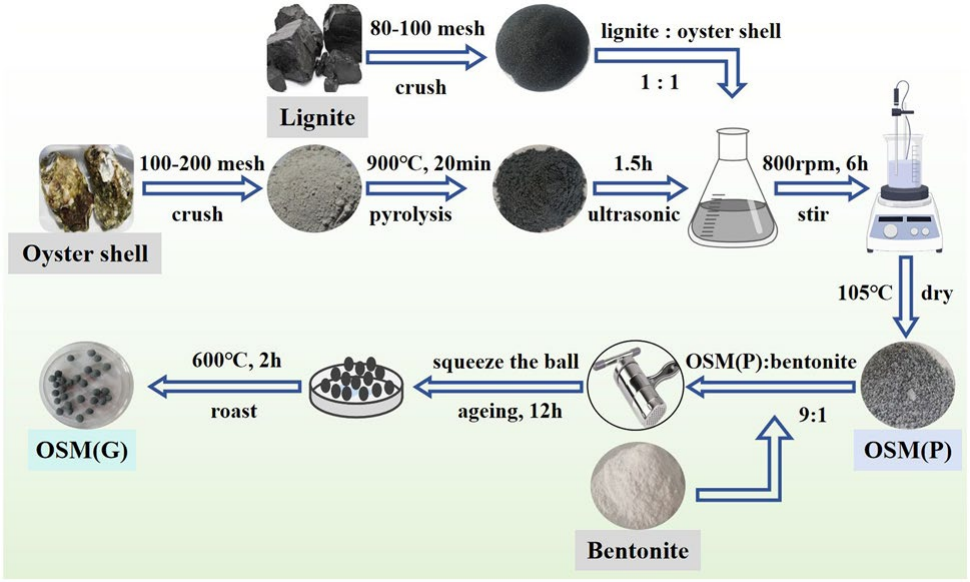
Preparation process of OS-M(P) and OS-M(G).

The preparation steps for OS-M(P) and OS-M(G) are shown in Fig. 1.

Preparation of OS-M(P): 5 g of oyster shells were placed in a crucible, placed in a muffle furnace and pyrolyzed at 900 °C for 20 min. The pyrolyzed oyster shells were added to 250 mL of deionized water and treated with ultrasound for 1.5 h to obtain a pyrolyzed oyster shell suspension. The mixture was obtained by adding 5 g of lignite to the oyster shell suspension in a mass ratio of 1:1 and stirring at 800 rpm for 6 h in a room temperature magnetic stirrer. The mixture was transferred to an oven at 105 °C for drying in order to remove the moisture and to obtain the OS-M(P) composite.

Preparation of OS-M(G): OS-M(P) and bentonite binder were mixed at a ratio of 9:1, a certain amount of deionized water was added, and the mixture was kneaded repeatedly and thoroughly until it was uniform and neither hard nor thin and soft, and a small ball of about 3–5 mm was made by squeezing the ball and sealed for 12 h. The aged pellets were put into a crucible, placed in a muffle furnace preheated to 250 °C, then heated to 600 °C and roasted for 2 h, and then cooled naturally after removal to obtain OS-M(G) composites.

### Batch adsorption experiments

The removal rate of Pb(II) and Cd(II) and the regulation efficiency of acidity of OS-M(P) and OS-M(G) were evaluated by batch experiments. The effects of various factors on the adsorption properties of OS-M(P) and OS-M(G) were investigated. The factors include the adsorbent dosage (Experiment 1), solution pH value (Experiment 2), adsorption time (Experiment 3), initial solution concentration and temperature (Experiment 4) in a monadic metal system, and the initial solution concentration in binary metal system (Experiment 5). Batch adsorption experiment parameters are shown in Table 1. All adsorption experiments were repeated in 150 mL conical bottles, and OS-M(P) and OS-M(G) were added to simulated AMD containing Pb(II) and Cd(II), respectively, and shaken at 120 rpm on a horizontal bench top shaker. To detect the concentration of Pb(II) and Cd(II) in the solution after the reaction, the samples were centrifuged at 4000 g for 10 min, and the supernatant was filtered through a 0.45 μm membrane. The removal rate (*R*_e_), adsorption capacity at time t (*q*_t_), and equilibrium adsorption capacity (*q*_e_) were calculated, respectively.

**Table 1:** Summary of parameters for the batch adsorption experiments.

Conditions	Adsorbent dosage (g/L)	Adsorption time (min)	pH	Initial concentration (mg/L)	Temperature (°C)
Monadic metal system					
Experiment 1	0.05, 0.1, 0.2, 0.3 and 0.5 for OS-M(P) 1.0, 2.0, 3.0, 4.0, 5.0 and 6.0 for OS-M(G)	720	4	50 for Pb(II); 10 for Cd(II)	25
Experiment 2	0.2 for OS-M(P); 2.0, 4.0 for OS-M(G)	720	2, 3, 4, 5, 6	50 for Pb(II); 10 for Cd(II)	25
Experiment 3	0.2 for OS-M(P); 2.0, 4.0 for OS-M(G)	5, 10, 15, 30, 60, 120, 240, 360, 480, 600 and 720	4	50 for Pb(II); 10 for Cd(II)	25
Experiment 4	0.2 for OS-M(P); 2.0, 4.0 for OS-M(G)	720	4	10, 30, 50, 70 and 100 for Pb(II) 10, 30, 50, 70 and 100 for Cd(II)	15, 25, 35
Binary metal system					
Experiment 5	0.2 for OS-M(P); 2.0, 4.0 for OS-M(G)	720	4	Pb–Cd: 10, 30, 50, 70 and 100 for Pb(II); 10 for Cd(II) Cd–Pb: 10, 30, 50, 70 and 100 for Cd(II); 50 for Pb(II)	25

The adsorption kinetics, isotherm and thermodynamic behavior of Pb(II) and Cd(II) adsorbed by OS-M(P) and OS-M(G) were investigated. Pseudo-first order (PFO), pseudo-second order (PSO) and intra-particle diffusion (IPD) kinetics were used to fit the kinetic data. Langmuir and Freundlich models were used to fit the adsorption isotherm data. Gibbs free energy Δ*G*, entropy change Δ*H* and enthalpy change Δ*S* were used to describe the thermodynamic behavior.

### Heavy metal leaching and regeneration experiments

The saturated OS-M(P) and OS-M(G) containing Pb(II) and Cd(II) were washed several times with deionized water and dried in an oven at 80 °C for 24 h. 1000 mL of distilled water was prepared, pH was adjusted to the range of 3–11 with HNO_3_ and NaOH solution, and the desorption solution was obtained. 0.2 g dried OS-M(P) and 4 g dried OS-M(G) were added to the desorption solution and shaken on a shaking table at 25 °C and 150 rpm for 2,4,6,8,10,12 h. After desorption, the supernatant was filtered through a 0.45 μm membrane, and the sample was centrifuged at 4000 g for 10 min. The concentrations of Pb(II) and Cd(II) in the solution were measured, and the desorption rate *η* (%) was calculated.

In order to investigate the reusability of OS-M(P) and OS-M(G), the desorption solution with the best pH above was selected for the regeneration experiment. The separated OS-M(P) and OS-M(G) are washed to remove the desorption solution and dried at 80 °C until a constant weight *M*_x_ is reached. The adsorbent was added to the simulated AMD for the adsorption experiment. After adsorption, the adsorbent was added to the desorption solution again for the second adsorption–desorption cycle experiment. And so on, the experiment was carried out for a total of 5 adsorption–desorption cycles. The removal rate of Pb(II) and Cd(II) and the quality of the adsorbent were measured in each regeneration experiment.

### Application of adsorbents in actual AMD treatment

The actual AMD samples were collected from the tailings water of a lead–zinc mine in Huludao City, Liaoning Province, China. Samples were collected in 5 L polypropylene bottles, kept at a constant temperature of 4 °C and tested on the day of collection. 0.2 g/L OS-M(P) and 4 g/L OS-M(G) were added to the actual AMD wastewater, and the reaction conditions were consistent with the adsorption experiment. The adsorption capacity of the adsorbent for pH, heavy metals (Pb, Cd, Fe, Mn, Cu, and Zn) and anion (SO_4_^2−^) in the wastewater was determined.

### Characterizations and analysis

Brunner-Emmet-Teller (BET, Micromeritics ASAP 2020, USA) N_2_ adsorption and desorption method was used to measure the specific surface area, total pore volume and mean pore size of the materials. he surface morphology and metal distribution of the materials were examined by scanning electron microscope (SEM, Regulus 8100, Japan). Elemental mapping was completed by energy dispersive spectroscopy (EDS, Thermo ScientificTM Ultra Dry, USA). The phase composition and crystal structure of the materials were studied by X-ray diffraction (XRD, Bruker D8 advance, Germany). XRD is used to scan the material at a rate of 6°/min using monochromatic Cu/Kα rays (λ = 0.154 nm) at 40 kV and 30 mA operating conditions, and the scanning angle 2*θ* is 5°–90°. Fourier transform infrared spectroscopy (FTIR, Thermo ScientificTM Nicolet iS10, USA) was used to characterize the surface functional groups of the materials. The wave number ranges from 400 to 4000 cm^−1^.

The pH of the wastewater was determined by the glass electrode method (GB/T 6920-86). Concentrations of heavy metal (Pb, Cd, Zn, Cu) were measured by atomic absorption spectrometer method (GB 7475-87). The wavelengths used for the analysis of the Pb, Cd, Zn, and Cu were 283.3, 228.8, 213.8, and 324.7 nm, respectively. Heavy metal ions (Fe and Mn) were determined by the flame atomic absorption spectrophotometry method (GB 11911-89). The wavelengths used for the analysis of the Fe and Mn were 248.3, and 279.5 nm, respectively. The SO_4_^2−^ in wastewater was determined by the barium chromate spectrophotometry (HJ/T 342-2007).

## Results and discussion

### Characteristics of OS-M(P) and OS-M(G)

#### Pore structure of OS-M(P) and OS-M(G)

**Figure 2:**
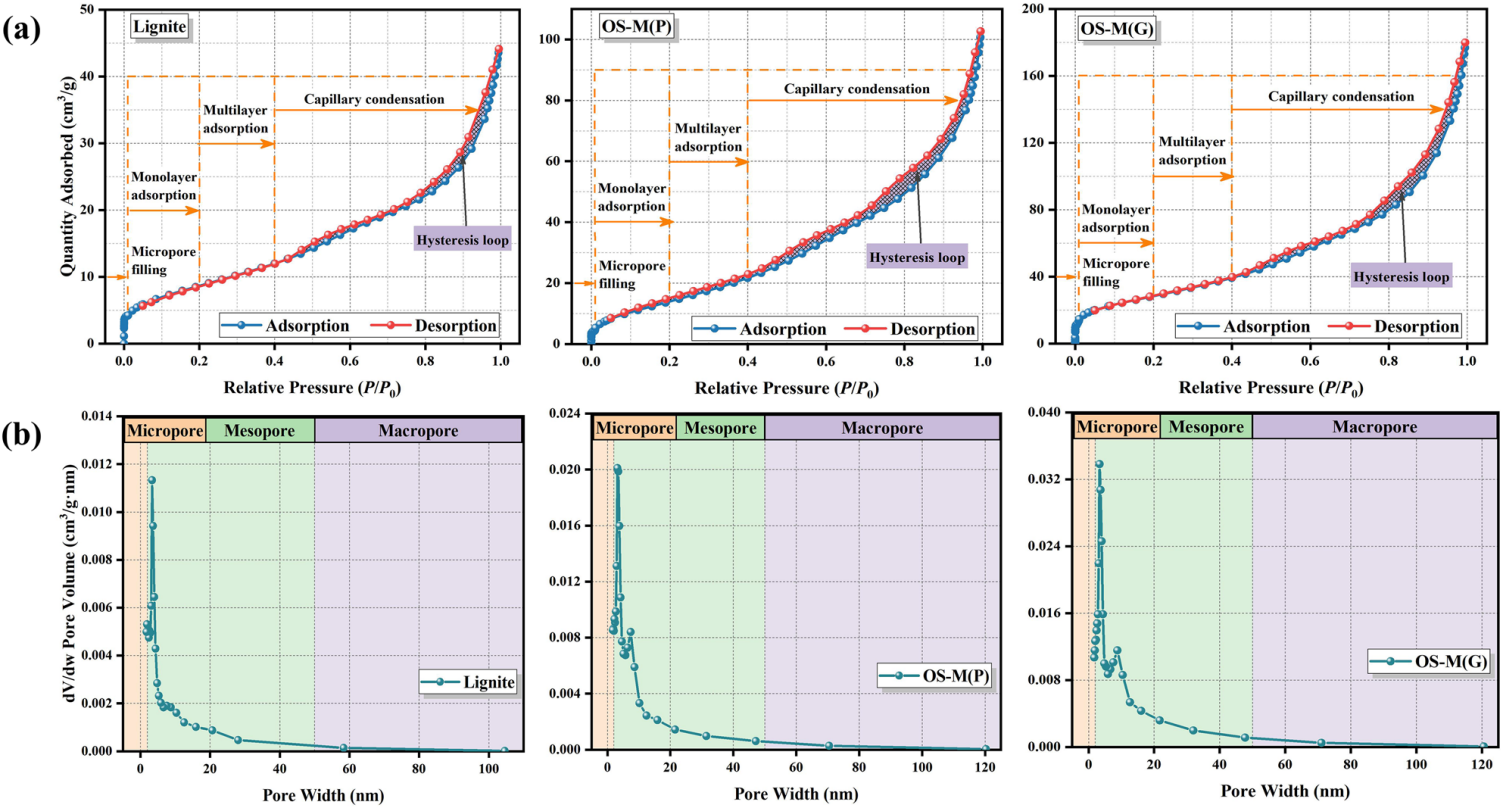
BET results of lignite, OS-M(P) and OS-M(G). (**a**) N_2_ adsorption–desorption isotherm. (**b**) Pore diameter distribution.

The BET analysis results of lignite, OS-M(P) and OS-M(G) are shown in Fig. 2. Figure 2a shows the N_2_ adsorption–desorption isotherm. The adsorption capacities of lignite, OS-M(P) and OS-M(G) were 44.122 cm^3^/g, 102.676 cm^3^/g and 179.948 cm^3^/g, respectively. The adsorption isotherm types show the same trend under different relative pressures, which is consistent with the typical type IV^47^. Under different relative pressures, there are four adsorption states: micropore filling, monolayer adsorption, multilayer adsorption and capillary condensation. The micropore filling phase is short, and the initial phase of the adsorption isotherm increases dramatically at very low relative pressure (*P*/*P*_0_ < 0.01). With the complete filling of the adsorbent on the micropores, the adsorbent molecules will form a single molecular layer on the entire surface of the adsorbent, and the adsorption isotherm shows a unique “knee” character (*P*/*P*_0_ = 0.01–0.2). When *P*/*P*_0_ = 0.2–0.4, further multimolecular adsorption occurred on the adsorbent surface. The surface of the adsorbent continued to be adsorbed in multiple layers, and the adsorption curve began to rise slowly, accompanied by capillary condensation (*P*/*P*_0_ > 0.4), and there was an obvious hysteresis loop in this stage^48^. The three adsorbents all conform to the typical H3 hysteresis loop^49^, indicating that they are mainly composed of slit pores formed by the accumulation of sheet particles. Figure 2b shows the aperture distribution. Lignite, OS-M(P) and OS-M(G) are mainly mesoporous (2–50 nm), and the pore sizes are mainly concentrated in the range of 3–10 nm. Table 2 shows the pore structure parameters of lignite, OS-M(P) and OS-M(G). The characteristics of surface area, pore size and pore volume of lignite after loading with oyster shell are changed to some extent. The order of the specific surface area and total pore volume of the three adsorbents was OS-M(G) > OS-M(P) > lignite, indicating that the adsorbents of the two forms have greater adsorption potential than lignite. The specific surface area of OS-M(G) increases because bentonite will lose surface water, bound water in the skeleton structure, and organic pollution in the pores in turn under the condition of high temperature calcination, resulting in increased porosity, looser structure, larger specific surface area, and certain improvement in adsorption properties. By comparing the pore sizes of the three adsorbents, it can be seen that OS-M(P) has more microporous structures than lignite, while OS-M(G) has more mesoporous or macroporous structures than lignite.

**Table 2:** Specific surface area, pore volume and pore diameter parameters of lignite, OS-M(P) and OS-M(G).

Parameters	lignite	OS-M(P)	OS-M(G)
Specific surface area (m^2^/g)	32.0523	55.0809	103.9347
pore volume (cm^3^/g)	0.0667	0.1549	0.2765
pore diameter (nm)	8.0304	7.8464	8.3334

#### Surface characteristics of OS-M(P) and OS-M(G)

**Figure 3:**
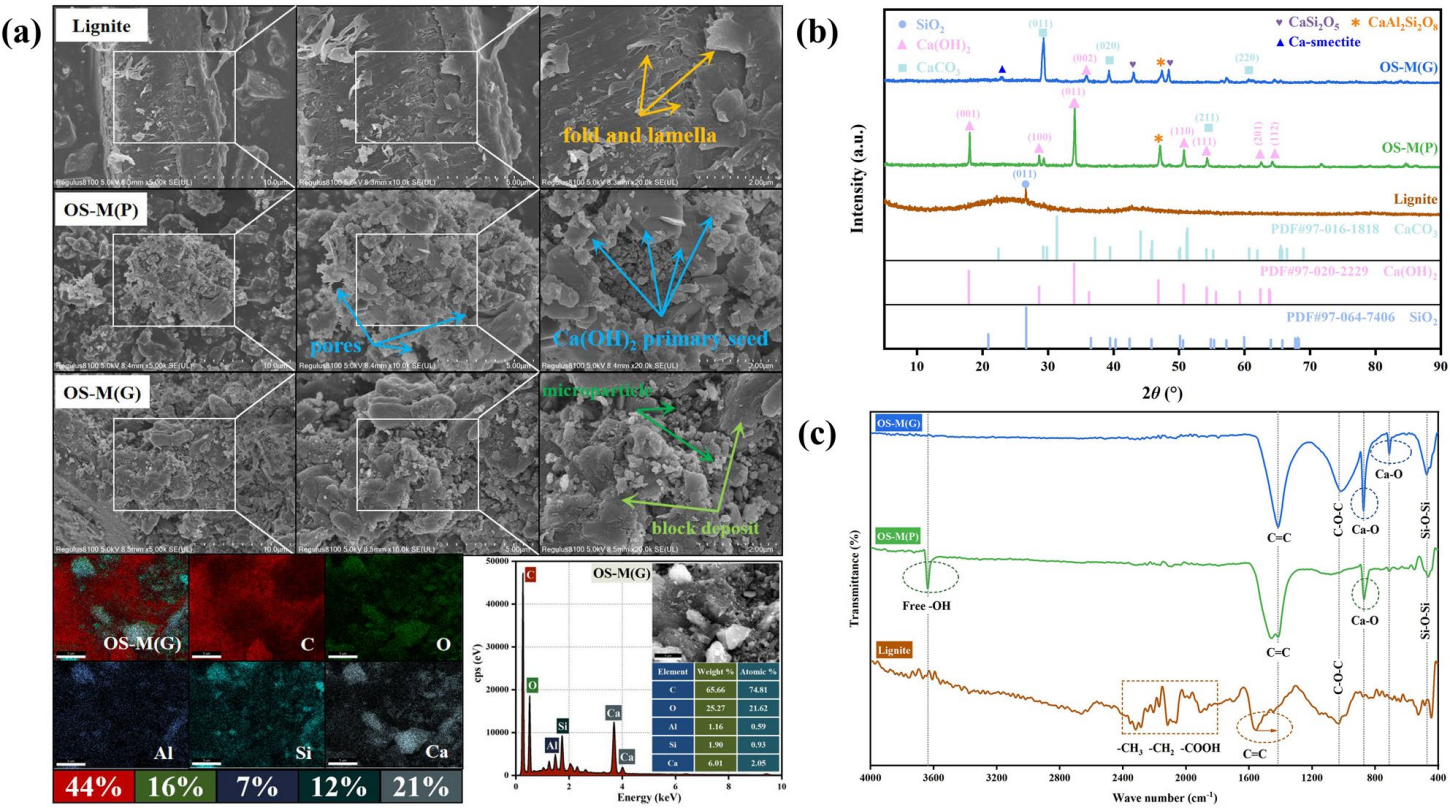
Microscopic characterization results of lignite, OS-M(P) and OS-M(G). (**a**) SEM–EDS. (**b**) XRD. (**c**) FTIR.

Figure 3a shows the SEM and EDS spectra of lignite, OS-M(P) and OS-M(G). The surface of lignite is relatively smooth and dense, but there are also some accumulation of micropores and lamellae on the surface, with a certain structure of folds and voids^29,50^. The surface of OS-M(P) is rougher than that of lignite, with many irregular pore structures. This is because oyster shells are more alkaline after calcination and ultrasonic treatment and corrode the surface of lignite. A large number of layered structures appear on the surface of OS-M(P), and some granular, mycelial and hexagonal structures are observed on the surface of OS-M(P) compared to lignite at 20 k magnification. According to the study of Khan et al.^51^ and Feng et al.^52^, these granular and mycelial structures are the primary crystal seeds of hexagonal crystal calcium hydroxide, which can rapidly grow into calcium hydroxide crystals, connect and accumulate on the surface of OS-M(P) to form a large number of layered structures, increasing its surface area. The surface roughness of OS-M(P) increases, the number of pores increases, the surface area increases, and more adsorption sites are exposed, which is conducive to the adsorption of heavy metals. There are many cracks, pores and scaly structures on the surface of OS-M(G), and a large number of fine particles, flake structures and massive structures accumulate. There are three causes of this phenomenon. First, high-temperature roasting leads to the release of water and some organic matter in lignite, the pore structure shrinks and collapses, and the surface roughness increases. Second, the scale structure of the bentonite binder is retained, making OS-M(G) loose and porous. Third, oyster shells are decomposed into CaO and CO_2_ after roasting. This analysis is also supported by the significant reduction in the C and O content of OS-M(G) compared to lignite. A large amount of CO_2_ will play a role in opening the pores, so that the internal pores of OS-M(G) will increase. At the same time, the CaO generated by high-temperature roasting will enter the bentonite and lignite layers and combine with SiO_2_, silicate precursors or aluminosilicate precursors to form C–S–H, C–A–H, C–A–S–H and other crystals or amorphous gels, which are deposited in the form of fine particles, flake structure or block structure to increase the surface area of the material^53,54^. The EDS energy spectrum shows that large amounts of C, O, Al, Si and trace amounts of Ca are mainly distributed on the surface of lignite. The content of C in OS-M(P) and OS-M(G) decreases significantly, the content of Ca increases significantly, and the change of O, Al and Si is not significant.

#### Phase composition of OS-M(P) and OS-M(G)

Figure 3b shows the XRD patterns of lignite, OS-M(P) and OS-M(G). Lignite has an obvious characteristic peak at 26.603°, which is the (003) plane diffraction peak of carbon, confirming that lignite has an amorphous carbon structure^53^. The (011) plane diffraction peak of SiO_2_ appears at 26.639°, indicating that the minerals of lignite are composed of quartz^55^. The characteristic peaks of Ca(OH)_2_ and CaCO_3_ in OS-M(P) and OS-M(G), respectively, indicate the successful loading of oyster shells on lignite. Oyster shells rich in CaCO_3_ are decomposed to CaO after calcination at high temperature, and part of CaO is hydrolyzed to Ca(OH)_2_ after calcination and ultrasonic treatment. The characteristic peaks of SiO_2_ in OS-M(P) and OS-M(G) lignite disappear because part of SiO_2_ participates in the volcanic ash reaction to form CaSi_2_O_5_ and CaAl_2_Si_2_O_8_ mineral phases, and the other part of SiO_2_ becomes an amorphous gel. The main component of Ca-bentonite in OS-M(G) is dioctahedral type Ca-smectite at 22°. Due to the incomplete dehydroxylation stage at 600 °C, part of the Ca-smectite structure is decomposed, but the incomplete decomposition can still be observed^56^.

#### Functional groups on the surface of OS-M(P) and OS-M(G)

Figure 3c shows the FTIR spectra of lignite, OS-M(P) and OS-M(G). The Si–O-Si characteristic absorption peaks appeared in the vicinity of 470 cm^−1^, indicating that the Si–O-Si structure was not destroyed during high-temperature pyrolysis and roasting, but the characteristic absorption peaks were significantly enhanced in OS-M(G), which also verified the appearance of new silicates and silicon oxides. In the vicinity of 1450 cm^−1^ and 1050 cm^−1^, the skeletal stretching vibration and the C–O–C stretching vibration, representing the C=C aromatic ring of lignite structure, appeared, respectively^57^. Compared with lignite, the –CH_2_ and –CH_3_ stretching vibration peaks between 1800 and 2400 cm^−1^ in OS-M(P) and OS-M(G) become weaker and the –COOH characteristic peaks disappear, indicating that some organic components in lignite may be decomposed after high-temperature pyrolysis and roasting. However, new characteristic absorption peaks appear near 870 cm^−1^ and 710 cm^−1^, which we speculate may be the bending vibration of Ca-O, indicating that oyster shells were successfully loaded on the surface of lignite through the combination of Ca-O bonds^58,59^. It is noteworthy that in OS-M(P), a strong and narrow band of free -OH bending vibration appears near 3640 cm^−1^, which provides the -OH supplied by Ca(OH)_2_.

### Batch adsorption experiments

#### The effect of adsorbent dosages

**Figure 4:**
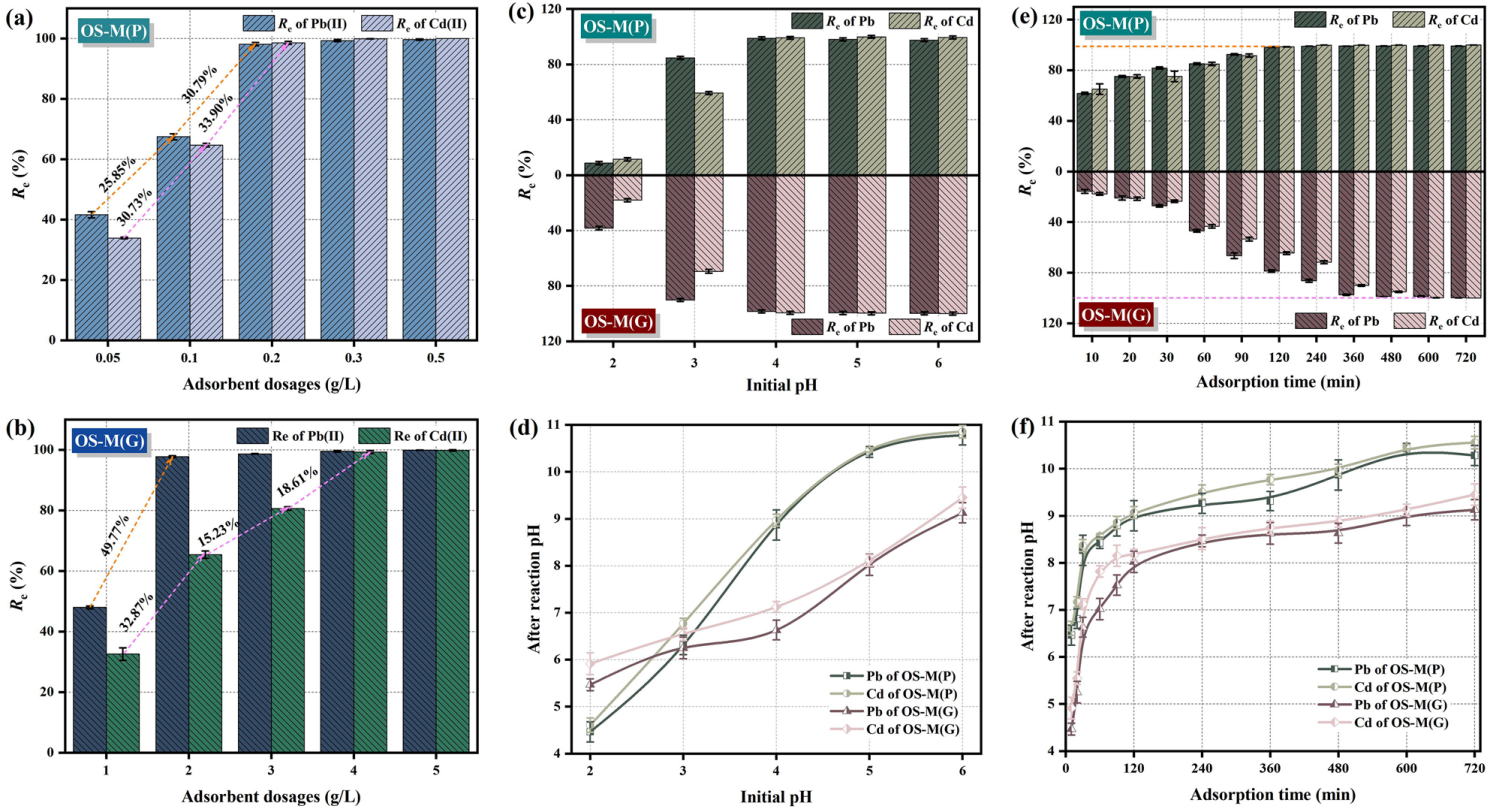

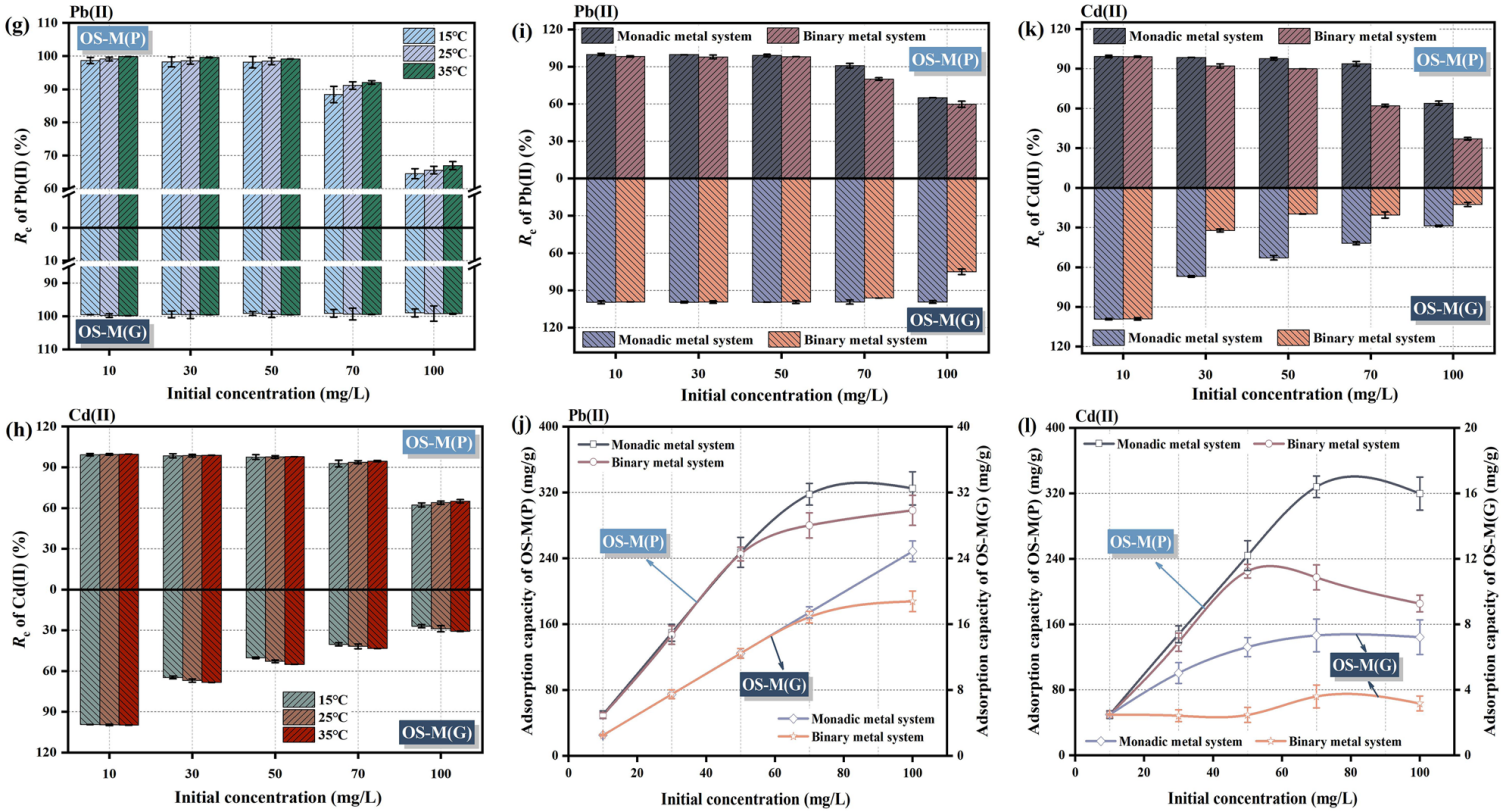
Adsorption batch experiment results of OS-M(P) and OS-M(G). (**a**, **b**) adsorbent dosages. (**c**, **d**) initial pH. (**e**, **f**) adsorption time. (**g**, **h**) Initial concentration. (**i**, **j**, **k**, **l**) Binary metal system.

Figure 4a,b shows the effect of adsorbent dosage on the adsorption of Pb(II) and Cd(II) by OS-M(P) and OS-M(G). It can be seen from Fig. 4a that when the dosage of OS-M(P) was increased from 0.05 to 0.2 g/L, its removal rate of Pb(II) and Cd(II) increased significantly because the increase of dosage provided more effective adsorption sites. The removal rate of Pb(II) and Cd(II) increased slightly, but not significantly, when the dosage was further increased to 0.5 g/L. It may be that excessive powder adsorbent is agglomerated, the effective adsorption site is reduced, and the utilization rate of adsorbent is limited. Therefore, the optimum dosage of OS-M(P) is 0.2 g/L. It can be seen from Fig. 4b that when the dosage of OS-M(G) is in the range of 1–2 g/L, its removal rate of Pb(II) increases significantly, indicating that the adsorption site of OS-M(G) can be fully utilized. The efficiency of increasing the removal rate of Pb(II) is not high, so the optimal dosage of OS-M(G) adsorption of Pb(II) is 2 g/L. When the dosage of OS-M(G) reached 4 g/L, the removal rate of Cd(II) increased the most. Therefore, the optimum dosage of OS-M(G) adsorption Cd(II) is 4 g/L. The difference in dosage required for the effective removal of Pb(II) and Cd(II) by OS-M(G) is related to the difference in radius, hydration energy and solubility product constant of Pb(II) and Cd(II). Compared with Cd(II), Pb(II) has a smaller radius, hydration energy and solubility product constant, and is more prone to precipitation, ion exchange and complexation reactions.

Compared with the amount of adsorbent required by OS-M(P) and OS-M(G) to remove Pb(II) and Cd(II), it can be seen that the dosage of OS-M(P) is smaller, which has obvious advantages in saving material cost. OS-M(G) has a larger particle size and a smaller surface area than OS-M(P), and a larger dosage is required to provide more active adsorption sites, but this will reduce the adsorption capacity per unit mass of OS-M(G).

#### The effect of pH

Figure 4c,d shows the effect of initial pH value on the adsorption of Pb(II) and Cd(II) by OS-M(P) and OS-M(G). It can be seen from Fig. 4c that as the initial pH value of the solution increases from 2 to 4, the removal rates of Pb(II) and Cd(II) by OS-M(P) and OS-M(G) both increase significantly. As the pH value continues to increase from 4 to 6, the removal rate curve gradually becomes stable. The initial pH value of the solution system affects the electrostatic interaction between Pb(II) and Cd(II) and the adsorbent, surface precipitation and complexation reaction, and other physicochemical adsorption reactions^60^. The initial pH of the solution is low, and a large number of H^+^ will occupy the adsorption site, causing competitive adsorption with Pb(II) and Cd(II). At the same time, the OH^−^, Pb(II) and Cd(II) released by the adsorbent will be consumed and the chance of hydroxide precipitation will be reduced. When the initial pH of the solution is increased, the concentration of H^+^ is low, which is conducive to weakening the competitive adsorption of H^+^ with Pb(II) and Cd(II), and more adsorption sites are left on the surface of the adsorbent for the adsorption of target pollutants. At the same time, the low concentration of H^+^ will increase the negative charge on the surface of the adsorbent, reduce the electrostatic repulsion, and promote the surface precipitation of Pb(II) and Cd(II) with OH^−^ or combine more OH^−^ in the form of multiple basic ions^61^. It can be seen from Fig. 4d that OS-M(P) and OS-M(G) both release alkalinity and regulate the pH of the solution. If the initial pH of the solution is in the range of 2 to 4, the pH of the solution after the reaction is increased and within 9, and if the initial pH of the solution is more than 4, the pH of the solution is more than 9 or even 10, and the pH of the water body is more than 10, which will cause alkali pollution in the water body and the instability of pollutant precipitation.

Compared with the suitable pH range for the adsorption of Pb(II) and Cd(II) by OS-M(P) and OS-M(G), it can be seen that their suitable range for the treatment of AMD is within 2 ~ 4. In addition, the pH value of the solution will affect the adsorption reaction mechanism, and the stability of the compounds produced by different reaction mechanisms is different. When pH < 7, Pb(II) and Cd(II) exist in ionic form, and the removal of metal ions by adsorbent is mainly based on ion exchange and surface complexation. When the pH is between 7 and 9, Pb(II) and Cd(II) exist mainly in the form of Pb(OH)^+^, Cd(OH)^+^, Pb(OH)_2_ and Cd(OH)_2_, and the removal of metal by adsorbent is mainly by chemical precipitation. When pH > 9, Pb(II) and Cd(II) mainly exist in the form of Pb(OH)_2_, Pb(OH)_3_^−^, Pb(OH)_4_^2−^, Cd(OH)_2_, Cd(OH)_3_^−^, and the removal of metal by adsorbent is mainly based on chemical precipitation, but too high pH value will lead to secondary dissolution of hydroxide precipitation^62^. Therefore, under the condition of initial pH value of 4, considering the adsorption effect and product stability of adsorption reaction, OS-M(G) is more advantageous than OS-M(P) in treating Pb(II) and Cd(II) in AMD.

#### The effect of adsorption time

Figure 4e,f shows the effect of adsorption time on the adsorption of Pb(II) and Cd(II) by OS-M(P) and OS-M(G). It can be seen from Fig. 4e that the removal rates of Pb(II) and Cd(II) by OS-M(P) increased rapidly within 10–120 min, and the adsorption reaction gradually reached equilibrium after 120 min, at which time the removal rates of Pb(II) and Cd(II) were 98.32% and 98.58%, respectively. 120 min before adsorption, the adsorption rate of Pb(II) and Cd(II) by OS-M(G) increased rapidly. With the progress of the reaction, the adsorption of Pb(II) by OS-M(G) almost reached equilibrium at 480 min, and the removal rate of Pb(II) was 98.82%. At 600 min, the adsorption of Cd(II) by OS-M(G) reached equilibrium, and the removal rate was 99.83%. In the early stage of adsorption, the concentration of Pb(II) and Cd(II) in the solution is large, and a certain mass transfer driving force is generated under the large concentration pressure difference, which promotes the rapid adsorption of a large amount of Pb(II) and Cd(II) on the abundant adsorption sites on the surface of OS-M(P) and OS-M(G), so the adsorption efficiency is high and the adsorption speed is fast at this stage. With the continuous progress of the adsorption reaction, the surface adsorption sites of OS-M(P) and OS-M(G) are occupied by a large number of positively charged pollutants on the surface, and the electrostatic repulsion between them and the residual Pb(II) and Cd(II) in the solution reduces the adsorption reaction rate, so the adsorption reaches equilibrium at this stage. It can be seen from Fig. 4f that the alkalinity released by OS-M(P) and OS-M(G) increases with the increase of reaction time. At 120 min, after OS-M(P) adsorbed Pb(II) and Cd(II), the pH of the solution reached 9.00 and 9.07, respectively. After 480 min, the pH of OS-M(G) solution containing Pb(II) was increased to 9.13; after 600 min, the pH of OS-M(G) solution containing Cd(II) was increased to 9.21.

Compared with the time required for the adsorption of Pb(II) and Cd(II) and the increase in pH, it can be seen that OS-M(P) reaches the adsorption equilibrium faster because the addition of OS-M(P) to the solution releases alkalinity quickly, and Pb(II) and Cd(II) are mainly removed by hydroxide precipitation. The removal mechanism is simple, and the stability of the precipitate produced is poor. The adsorption process of Pb(II) and Cd(II) by OS-M(G) is slower because the addition of OS-M(G) to the solution will continue and slowly release basicity, which is conducive to continuous electrostatic adsorption, neutralization, precipitation, ion exchange or complexation reaction of OS-M(G) with Pb(II) and Cd(II) to form stable compounds for complete removal of pollutants.

#### The effect of initial concentration in different temperature

Figure 4g,h shows the effect of initial concentration on the adsorption of Pb(II) and Cd(II) by OS-M(P) and OS-M(G) at different temperature systems. It can be seen from Fig. 4g,h that with the increase of initial concentrations of Pb(II) and Cd(II) in the same temperature system, the removal rates of Pb(II) and Cd(II) by OS-M(P) and OS-M(G) gradually decrease. This is because at low concentrations, compared with the amount of pollutants in the solution, the number of effective adsorption sites on the surface of OS-M(P) and OS-M(G) is sufficient, which is conducive to Pb(II) and Cd(II) diffusing to the adsorption site and promoting the adsorption reaction. At high concentrations, the concentration pressure difference between the solution and the surface of the adsorbent continues to increase, which strongly drives the Pb(II) and Cd(II) in the solution to move rapidly to the surface of OS-M(P) and OS-M(G), increasing the chance of collision and interaction with the active site on the surface of the adsorbent. However, when the concentration is too high, the adsorbent gradually reaches saturation, and excessive positive charge will accumulate on the surface of the adsorbent after adsorption of Pb(II) and Cd(II), and the electrostatic repulsion will make it difficult to continue to adsorb more free metal ions in the solution, so the removal rate will gradually decrease^63^. It can also be seen from Fig. 4g,h that at the same concentration level, the removal rates of Pb(II) and Cd(II) by OS-M(P) and OS-M(G) increase with the increase of ambient temperature, indicating that the adsorption process of Pb(II) and Cd(II) is endothermic. As the temperature increases, the proportion of activated ions in the solution increases, Pb(II) and Cd(II) diffuse to the surface of the adsorbent at a faster diffusion rate, and the chance of effective collision with the functional groups on its surface increases, and the level of adsorption equilibrium increases^64^.

By comparing the removal rates of Pb(II) and Cd(II) at different initial concentrations of OS-M(P) and OS-M(G) in different temperature systems, it can be seen that the addition of 0.2 g/L OS-M(P) can treat the solution with the initial Pb(II) concentration of 0–50 mg/L, and the removal rate can reach more than 98%. The addition of 2 g/L OS-M(G) can treat the solution with initial Pb(II) concentration ranging from 0 to 100 mg/L, and the removal rate can reach more than 99%, and the Pb(II) in wastewater can be discharged up to the standard (Fig. 4g). The addition of 0.2 g/L OS-M(P) can treat the solution with the initial concentration of Cd(II) of 0–10 mg/L, and the removal rate can reach more than 99%, and the addition of 4 g/L OS-M(G) can treat the solution with the initial concentration of Cd(II) of 0–10 mg/L, and the removal rate can reach more than 99%, and the Cd(II) in wastewater can be discharged up to the standard (Fig. 4h). It can be seen that OS-M(G) can handle a wider range of concentrations containing Pb(II). Since the Cd content in acid mine drainage is generally lower and the Pb content is higher, OS-M(G) has certain advantages over OS-M(P) in treating Pb and Cd contamination in AMD.

#### The effect of binary competition system

Figure 4i–l shows the effect of binary metal system on the adsorption of Pb(II) and Cd(II) by OS-M(P) and OS-M(G). The removal rates and adsorption capacities of Pb(II) and Cd(II) in the binary metal system are lower than those in the mono-metal system, and the higher the initial concentration, the more significant the competition effect of Pb(II) and Cd(II). In the binary metal system, the total amount of metal ions in the solution is higher than that of the single system, so the higher the initial concentration, the greater the total amount of metal ions, the point site that can be provided by adding the same amount of adsorbent is limited, so the competitive adsorption between Pb(II) and Cd(II) ions is generated^65,66^. Overall, the removal rate and adsorption capacity of Pb(II) are higher than that of Cd(II), indicating that OS-M(P) and OS-M(G) are more selective for Pb(II) in the unequal competition system. Many scholars^67–69^ have found that the selectivity of adsorbents for heavy metal ions is jointly determined by the hydrolysis constant of metal ions, the radius of hydrated ions, the molar mass and the solubility product constant of metal ion hydroxide. The smaller the hydrolysis constant of metal ions, the easier the ions are adsorbed. The hydrolysis constant of Pb(II) (7.8) is significantly lower than that of Cd(II) (9.2). The smaller the hydration radius of the isovalent metal cation, the easier it is to diffuse through the boundary layer to the surface of the adsorbent, and the easier it is to undergo ion exchange reaction. The hydration radius of Pb(II) is smaller than that of Cd(II) (0.426 mm). The higher the molar mass of metal ions, the easier it is to precipitate and remove, and the molar mass of Pb(II) (207.2 g/mol) is almost twice that of Cd(II) (112.4 g/mol). The smaller the hydroxide solubility product of the isovalent metal ion, the easier it is to react with OH^−^ to produce chemical precipitation, and the hydroxide solubility product constant of Pb(II) (1.2 × 10^–15^) is smaller than that of Cd(II) (2.2 × 10^–14^). Therefore, it can be theoretically judged that OS-M(P) and OS-M(G) preferentially select Pb(II), which is also verified by the above experiments.

### Adsorption characteristic

#### Adsorption kinetics

**Figure 5:**
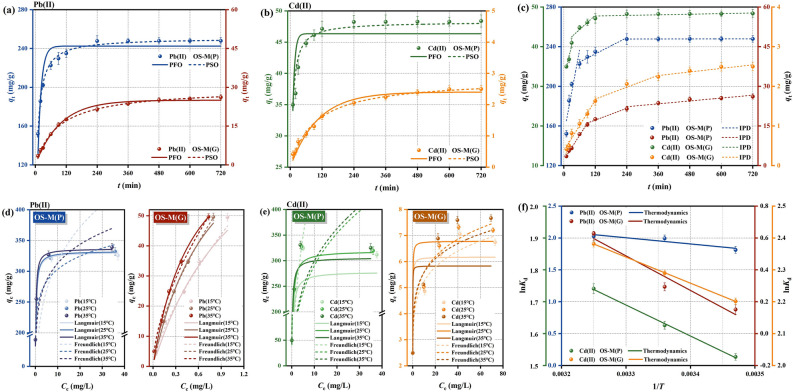
Adsorption characteristic of OS-M(P) and OS-M(G). (**a**) PFO and PSO models for Pb(II). (**b**) PFO and PSO models for Cd(II). (**c**) IPD models for Pb(II) and Cd(II). (**d**) Langmuir and Freundlich models for Pb(II). (**e**) Langmuir and Freundlich models for Cd(II). (**f**) Adsorption thermodynamics models for Pb(II) and Cd(II).

Adsorption kinetics is a means to study the adsorption process and adsorption rate. The adsorption kinetics of Pb(II) and Cd(II) by OS-M(P) and OS-M(G) are shown in Fig. 5a,b, and the kinetic fitting parameters are shown in Table 3. For the adsorption of Pb(II), the adsorption fitting of OS-M(P) and OS-M(G) was consistent with the pseudo-second-order kinetic model, and the correlation coefficient *R*^2^ (0.9956, 0.9959) was higher than that of the pseudo-first-order kinetic model (0.8396, 0.9919). Similar results were also found for the adsorption of Cd(II), and the *R*^2^ (0.9623, 0.9916) of the pseudo-second-order kinetic model was higher than that of the pseudo-first-order kinetic model (0.8150, 0.9763) (Table 3). The equilibrium adsorption capacities of OS-M(P) and OS-M(G) for Pb(II) were 242.4500 mg/g and 25.0033 mg/g, respectively, and the equilibrium adsorption capacities of Cd(II) were 46.3505 mg/g and 2.4031 mg/g, respectively. The pseudo-second-order kinetics is closer to the experimental results than the pseudo-first-order kinetics, so it is more suitable to explain the adsorption process of Pb(II) and Cd(II) by OS-M(P) and OS-M(G). It can be assumed that the adsorption rates of Pb(II) and Cd(II) by OS-M(P) and OS-M(G) are controlled by chemisorption mechanism, and the interaction forces between them are mainly van der Waals force and electrostatic force^70^. Using the intra-particle diffusion (IPD) model fitting Pb (II) and Cd (II) in OS-M(P) and OS-M(G) in the diffusion process of Fig. 5c. The adsorption process can be divided into three stages, namely surface film diffusion, intra-particle diffusion and adsorption equilibrium stage. In Table 3, the adsorption rate *k*_1_ > *k*_2_ > *k*_3_, and the boundary layer thickness *C*_1_ < *C*_2_ < *C*_3_ indicate that the increase of boundary layer thickness hinders the diffusion process of Pb(II) and Cd(II) to OS-M(P) and OS-M(G), and reduces the diffusion rate. The fitting equations of the three stages of the four curves in Fig. 5c do not pass through the origin, indicating that the total adsorption rates of Pb(II) and Cd(II) by OS-M(P) and OS-M(G) are jointly determined by membrane diffusion and intra granular diffusion^71^.

**Table 3:** The adsorption kinetics parameters of OS-M(P) and OS-M(G) for Pb(II) and Cd(II).

Kinetics model and parameters	Pb(II)		Cd(II)	
	OS-M(P)	OS-M(G)	OS-M(P)	OS-M(G)
Pseudo-first-order (PFO)				
K_1_	0.0748	0.0105	0.1327	0.0966
q_e_	242.4500	25.0033	46.3505	2.4031
R^2^	0.8396	0.9919	0.8150	0.9763
Pseudo-second-order (PSO)				
K_2_	0.0006	0.0004	0.0051	0.0039
q_e_	250.6846	29.5866	48.2535	2.8293
R^2^	0.9956	0.9959	0.9623	0.9916
Intra particle diffusion (IPD)				
k_1_	1.4216	0.1553	0.2675	0.0107
C_1_	151.1172	2.1232	32.2140	0.3688
R_1_^2^	0.7124	0.9891	0.8984	0.9659
k_2_	0.1358	0.0370	0.0603	0.0025
C_2_	216.4321	12.7888	40.6906	1.3768
R_2_^2^	0.9365	0.9183	0.7420	0.8670
k_3_	0.0004	0.0091	0.0013	0.0007
C_3_	247.6231	20.1115	47.5809	2.0045
R_3_^2^	0.7155	0.8675	0.9439	0.8235

#### Adsorption isotherm

Adsorption isotherm can effectively determine the distribution of adsorbent in the solid–liquid phase. The adsorption kinetics of Pb(II) and Cd(II) by OS-M(P) and OS-M(G) are shown in Fig. 5d, e, and the kinetic fitting parameters are shown in Table 4. The correlation coefficient (*R*^2^) of adsorption of Pb(II) and Cd(II) by OS-M(P) was compared. Langmuir model (0.9841–0.9972, 0.9595–0.9823) can better describe the adsorption process of Pb(II) and Cd(II) by OS-M(P) than Freundlich model (0.6613–0.7056, 0.7339–0.7882). The results show that the adsorption of Pb(II) and Cd(II) by OS-M(P) is mainly monolayer adsorption^72^. The maximum theoretical adsorption capacities *q*_m_ were 331.1064, 332.6219, and 336.6549 mg/g for Pb(II) and 278.4663, 318.9854, and 320.7048 mg/g for Cd(II), respectively, which were close to the experimental values. On the contrary, the correlation coefficient (*R*^2^) of adsorption of Pb(II) and Cd(II) by OS-M(G) was compared. Freundlich model (0.9792–0.9995, 0.9638–0.9887) can better describe the adsorption process of Pb(II) and Cd(II) by OS-M(G) than Langmuir model (0.9463–0.9949, 0.8822–0.9623). The results show that the adsorption of Pb(II) and Cd(II) by OS-M(G) is mainly based on the heterogeneous adsorption of multimolecular layers^72^. Table 5 compares the adsorption parameters of Pb(II) and Cd(II) with other powder morphology adsorbents. Obviously, compared with the adsorbents in the literature, the adsorption capacity of Pb(II) and Cd(II) was greatly improved by OS-M(P).

**Table 4:** The adsorption isotherm parameters of OS-M(P) and OS-M(G) for Pb(II) and Cd(II).

Isotherm model and parameters	Pb(II)						Cd(II)					
	OS-M(P)			OS-M(G)			OS-M(P)			OS-M(G)		
	15 °C	25 °C	35 °C	15 °C	25 °C	35 °C	15 °C	25 °C	35 °C	15 °C	25 °C	35 °C
Langmuir												
q_m_	331.1064	332.6219	336.6549	101.3423	93.6463	85.4838	278.4663	318.9854	320.7048	6.1763	6.7822	5.8340
K_L_	5.6535	6.0358	7.4839	0.8167	1.2940	1.8875	2.7857	2.7885	4.6931	11.4101	13.8031	39.3041
R^2^	0.9841	0.9972	0.9970	0.9463	0.9897	0.9949	0.9823	0.9659	0.9595	0.9602	0.9623	0.8822
Freundlich												
K_F_	185.2500	242.1503	229.6895	47.1470	57.3174	59.7620	175.1987	150.5919	158.2080	3.7478	3.9763	4.1319
1/n	0.2322	0.0976	0.1344	0.7010	0.6507	0.5938	0.4255	0.2847	0.2767	0.1452	0.1478	0.1307
R^2^	0.6952	0.6613	0.7056	0.9792	0.9995	0.9949	0.7882	0.7339	0.7471	0.9878	0.9887	0.9638

**Table 5:** Comparison of the maximum adsorption capacity of Pb(II) and Cd(II) with other adsorbents in powdery morphology.

Adsorbent	Adsorbate	Temperature (°C)	Time	Adsorption capacity (mg/g)	BET surface area (m^2^/g)	References
KMnO_4_-treated magnetic biochar (FMBC)	Pb(II)	25	24 h	148	137	Sun et al.^60^
	Cd(II)			79		
CA-enriched biochar of Chickpea	Pb(II)	25	24 h	12.1094	–	Nazari et al.^73^
	Cd(II)			2.4757		
Cassava root husk-derived biochar with ZnO nanoparticles (CRHB-ZnO_3_)	Pb(II)	25	60 min	44.27	2.7964	Tho et al.^74^
	Cd(II)			42.05		
Amine-functionalized magnesium ferrite-biochar composite (MgFe_2_O_4_–NH_2_@sRHB)	Pb(II)	25	24 h	198.93	51.30	Li et al.^75^
	Cd(II)			195.50		
Fe/S functionalized biochar (BC-Fe-S)	Pb(II)	45	120 min	124.62	41.30	Cao et al.^76^
	Cd(II)			57.71		
Mg/Fe bimetallic oxide-modified biochar	Pb(II)	25	480 min	283.4	332.84	Cheng et al.^77^
	Cd(II)			195.5		
Oyster shell loaded lignite composite adsorbent in powdery morphology (OS-M(P))	Pb(II)	25	720 min	332.62	102.676	This work
	Cd(II)			318.98		

#### Adsorption thermodynamics

The Gibbs free energy change (Δ*G*), enthalpy change (Δ*H*) and entropy change (Δ*S*) can determine the degree of difficulty of the adsorption process. With ln*K*_d_ as the vertical axis and 1/*T* as the horizontal axis, the trend plot of the dispersion distribution of thermodynamic changes was drawn and linear fit was performed. The results are shown in Fig. 5f, and the thermodynamic parameters are shown in Table 6. At temperatures of 288 K, 298 K and 308 K, Δ*G* of adsorption of Pb(II) and Cd(II) by OS-M(P) and OS-M(G) is negative, indicating that the adsorption process is spontaneous. The higher the temperature, the smaller the Δ*G*, and the more likely the spontaneous reaction occurs. The enthalpy change Δ*H* is positive, indicating that the adsorption reaction is an endothermic process. The entropy change Δ*S* is positive, indicating that the adsorption reaction is a process of entropy increase, the adsorbent and adsorbent coexist in the system, and the adsorption process of Pb(II) and Cd(II) is inevitably accompanied by the desorption of other ions at the adsorption sites on the surface of OS-M(P) and OS-M(G), which increases the chaos and disorder of the whole system.

**Table 6:** The adsorption thermodynamic parameters of OS-M(P) and OS-M(G) for Pb(II) and Cd(II).

	Δ*G*				
	15 °C	25 °C	35 °C	Δ*H*	Δ*S*
Pb(II)					
OS-M(P)	− 4.3411	− 4.9426	− 5.1909	7.9838	42.8814
OS-M(G)	− 0.3623	− 0.7286	− 1.6087	1.7500	61.7423
Cd(II)					
OS-M(P)	− 1.0843	− 1.2048	− 1.3278	7.8805	40.0290
OS-M(G)	− 5.2714	− 5.6734	− 6.0988	6.6392	41.3436

### Adsorption mechanism

**Figure 6:**
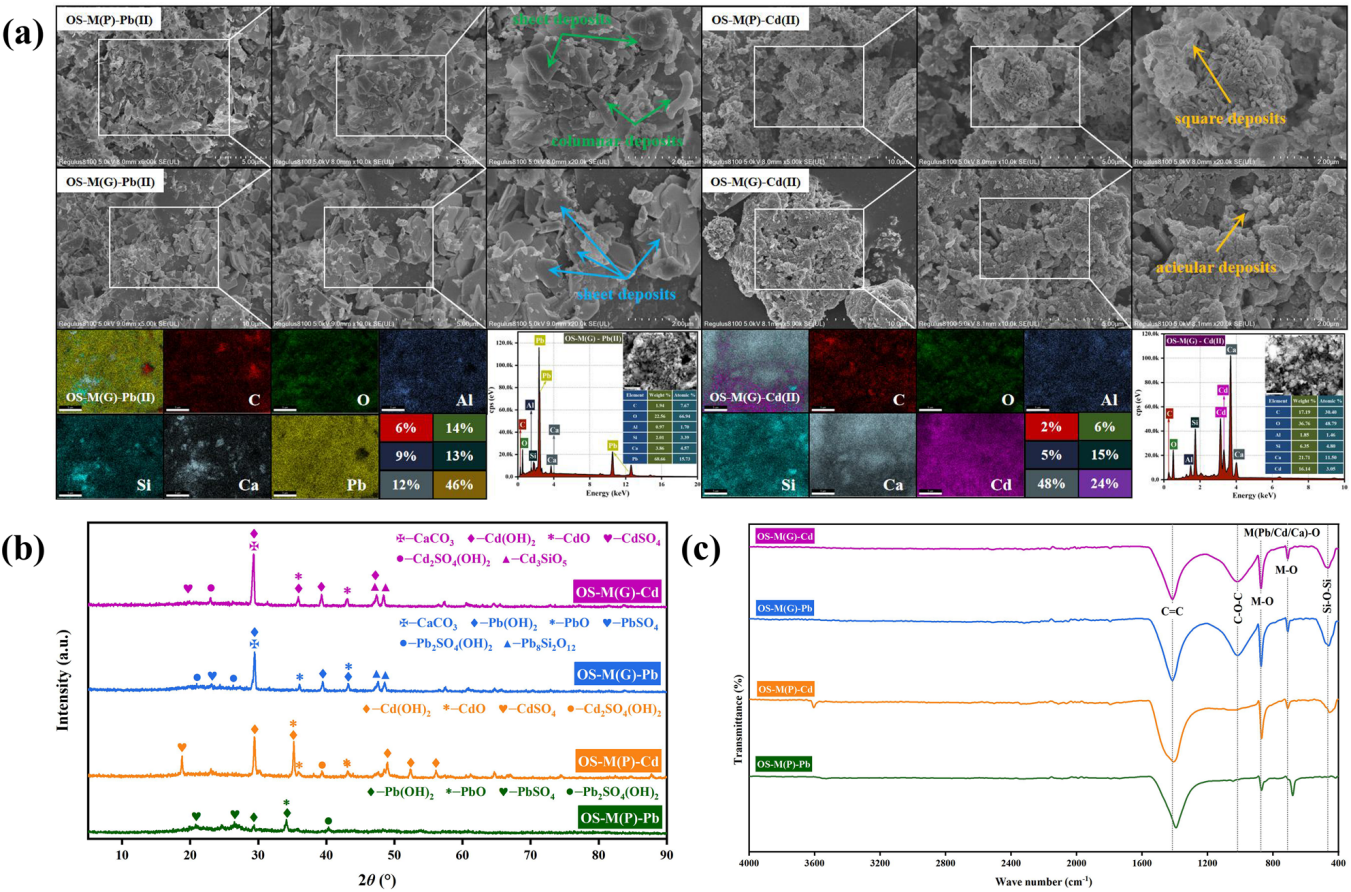
Microscopic characterization results for OS-M(P) and OS-M(G) after adsorption of Pb(II) and Cd(II). (**a**) SEM–EDS. (**b**) XRD. (**c**) FTIR.

Figure 6a shows the SEM and EDS spectra of Pb(II) and Cd(II) adsorbed by OS-M(P) and OS-M(P). After the adsorption of Pb(II) and Cd(II) by OS-M(P) and OS-M(G), the surface becomes rougher due to the accumulation of a large number of secondary solids generated by the surface precipitation reaction. They exist in the form of sheets, columns, squares and needles. It can be seen from the EDS energy spectrum, a large number of Pb and Cd elements obviously appear after adsorption, indicating that the adsorption sites of OS-M(P) and OS-M(G) successfully interact with Pb(II) and Cd(II) and generate stable products attached to them. The significant reduction of Ca content in the atlas can also confirm the occurrence of ion exchange reaction.

Figure 6b shows the XRD patterns of adsorption of Pb(II) and Cd(II) by OS-M(P) and OS-M(P). After adsorption of Pb(II) by OS-M(P), Pb(OH)_2_, PbO, PbSO_4_, Pb_2_SO_4_(OH)_2_ phases appeared. After OS-M(P) adsorbed Cd(II), Cd(OH)_2_, CdO, CdSO_4_ and Cd_2_SO_4_(OH) phases appeared. We speculated that Pb(OH)_2_ and Cd(OH)_2_ were formed by the chemical precipitation of OH^−^ provided by Ca(OH)_2_ on the surface of the adsorbent and Pb(II) and Cd(II) in AMD. This result was also confirmed by FTIR, which showed a weakening of the characteristic absorption peak at 3634 cm^−1^. PbO and CdO are oxides formed by thermal decomposition of Pb(OH)_2_ and Cd(OH)_2_. PbSO_4_ and CdSO_4_ are formed by ion exchange reactions between Pb(II) and Cd(II) and Mn(II) and Fe(II) provided by AMD mimicking drugs. When the pH of the solution is between 8 and 9, Pb(II) and Cd(II) exist as Pb(OH)^+^ and Cd(OH)^+^ in the alkaline solution and react with SO_4_^2−^ to form Pb_2_SO_4_(OH)_2_ and Cd_2_SO_4_(OH)_2_. After adsorption of Pb(II) and Cd(II) by OS-M(G), except CaCO_3_ phase still exists, the new phases are Pb(OH)_2_, PbO, PbSO_4_, Pb_2_SO_4_(OH)_2_, Pb_8_Si_2_O_12_ and Cd(OH)_2_, CdO, CdSO_4_, Cd_2_SO_4_(OH)_2_, Cd_3_SiO_5_. In addition to the same mechanism of adsorption of Pb(II) and Cd(II) with OS-M(P), it is worth noting that Pb_8_Si_2_O_12_ and Cd_3_SiO_5_ are thought to be produced by the ion exchange reaction of Pb(II) and Cd(II) with silicates in OS-M(G) or by electrostatic adsorption with free silicates in solution. Similar results were obtained by^34,54,78^.

Figure 6c shows the FTIR spectra of Pb(II) and Cd(II) adsorbed by OS-M(P) and OS-M(P). For OS-M(P), the free –OH near 3640 cm^−1^ was significantly weakened and shifted after the adsorption of Pb(II) and Cd(II), indicating that the functional group was involved in the adsorption process of Pb(II) and Cd(II) by OS-M(P). The C–O–C stretching vibration peak near 1050 cm^−1^ is slightly enhanced after adsorption, because C–O–C participates in the surface complexation of Pb(II) and Cd(II). The shift of the M–O absorption peak near 870 cm^−1^ and 710 cm^−1^ indicates that Pb(II) and Cd(II) have ion exchange reactions with Ca(II).

**Figure 7:**
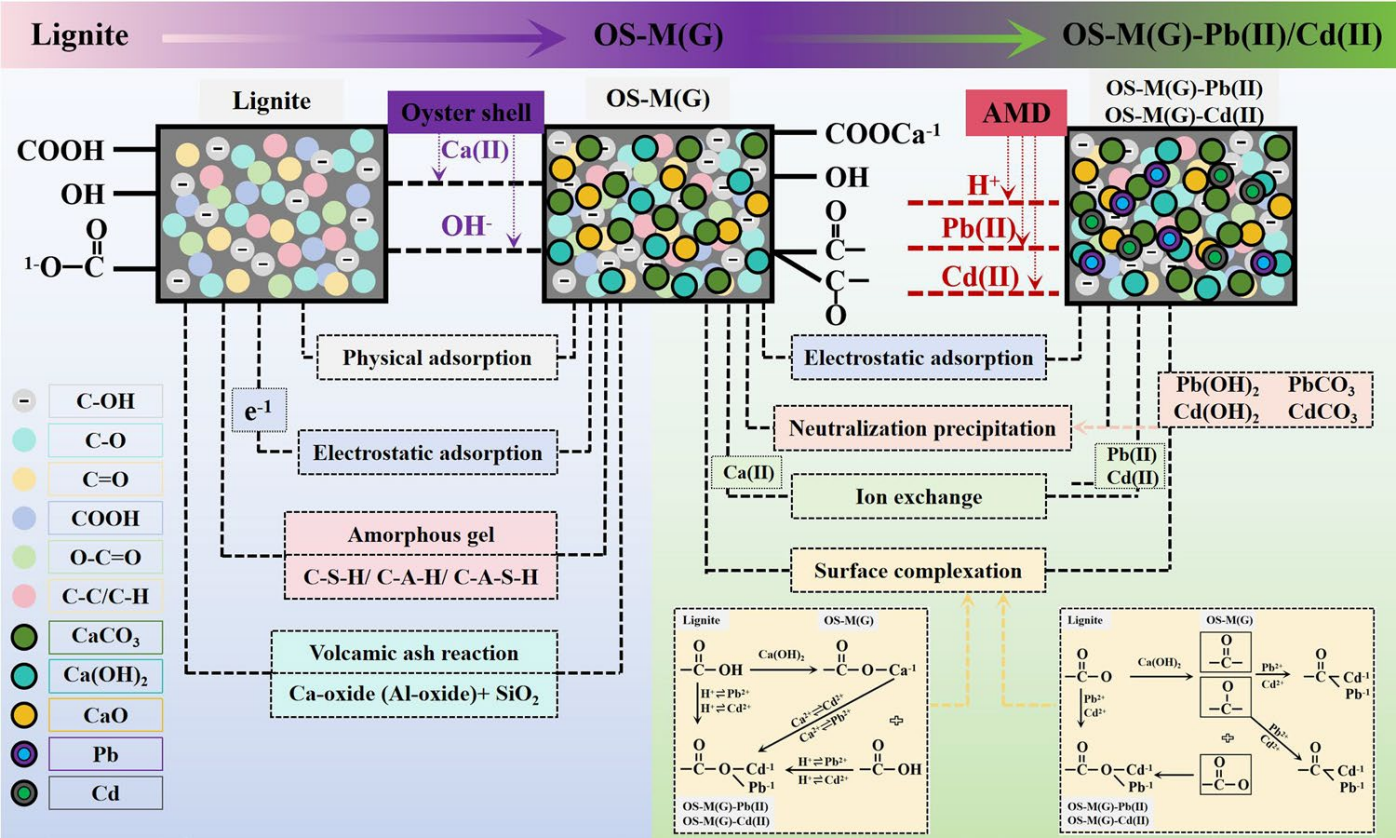
Revealed the adsorption mechanism of Pb(II) and Cd(II) in solution by lignite loaded with oyster shell. (taking OS-M(G) as an example).

It can be seen from the above adsorption characteristics and microscopic characterization results that OS-M(P) and OS-M(G) have the same adsorption mechanism for the adsorption of Pb(II) and Cd(II) in solution, which is mainly the result of the adsorption-coagulation synergism of electrostatic adsorption, neutralization precipitation, ion exchange and complexation reaction. Figure 7 shows the adsorption mechanism of Pb(II) and Cd(II) in solution by OS-M(G) as an example.

### Desorption, regeneration and application of adsorbents

**Figure 8:**
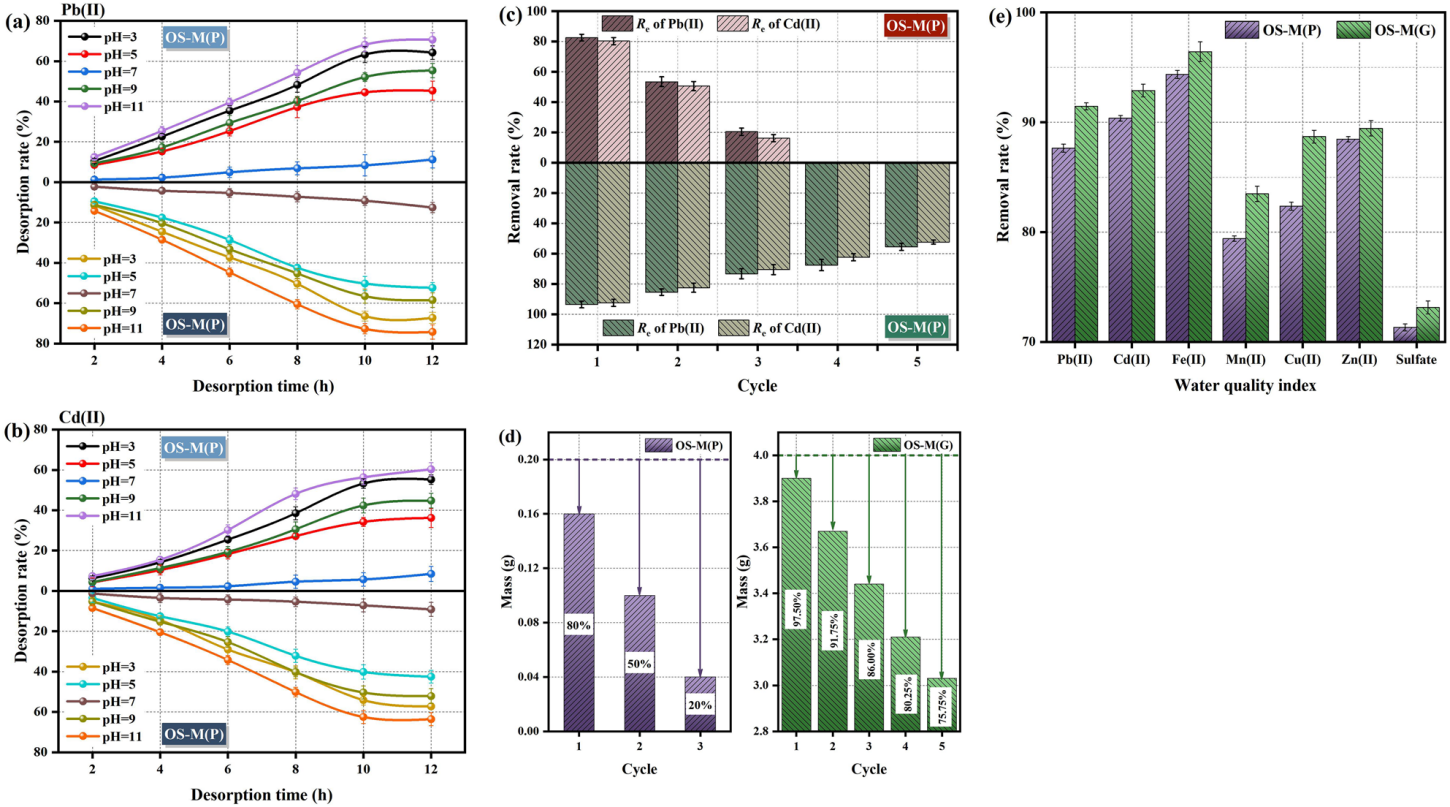
Experimental results of desorption, regeneration and application of adsorbents. (**a**, **b**) adsorption–desorption at different pH. (**c**) regeneration times of OS-M(P) and OS-M(G). (**d**) recovery rate of the adsorbents. (**e**) application in actual AMD.

At different pH levels, the desorption rates of Pb(II) and Cd(II) in saturated OS-M(P) and OS-M(G) are shown in Fig. 8a,b. At the initial stage, the desorption rates of Pb(II) and Cd(II) in the solution generally increased, and reached a stable level after 12 h. In addition, the pH of the solution can also significantly affect the release of heavy metals by saturated adsorbents. The desorption of Pb(II) and Cd(II) was better in acidic and alkaline solutions, and the desorption rate was the lowest in H_2_O solution. In acidic desorption solution (pH = 3), the desorption rates of Pb(II) and Cd(II) in OS-M(P) were 64.26% and 55.23%, respectively. The desorption rates of Pb(II) and Cd(II) in OS-M(G) were 67.21% and 57.21%, respectively. This may be because when the solution is acidic, there is an ion exchange reaction between the excess H^+^ in the solution and Pb(II) and Cd(II), and there is a competition for adsorption sites on the adsorbent. In alkaline desorption solution (pH = 11), the desorption rates of Pb(II) and Cd(II) in OS-M(P) were 70.65% and 60.29%L, respectively. The desorption rates of Pb(II) and Cd(II) in OS-M(G) were 74.26% and 63.57%, respectively. Because in the alkaline environment, the excess OH^−^ in the solution can form Pb(OH)_2_, [Pb(OH)_4_]^2−^, [Pb_3_(OH)_4_]^2+^ and Cd(OH)_2_ with Pb(II) and Cd(II), which is conducive to the desorption of Pb(II) and Cd(II). Therefore, saturated OS-M(P) and OS-M(G) have different regularities in Pb(II) and Cd(II) release at different pH levels.

Figure 8c shows the relationship between the regeneration times of OS-M(P) and OS-M(G) and the removal rate of heavy metals in the desorption solution at pH = 11, so as to evaluate the reusability of the adsorbent. After 5 times of adsorption and desorption, the removal rates of Pb(II) and Cd(II) by OS-M(G) were 55.47% and 52.36%, respectively, which were still at a high level, indicating that OS-M(G) had a high reusability value. However, after 3 sorption–desorption, the removal rates of Pb(II) and Cd(II) by OS-M(P) were 20.48% and 16.25%, respectively, and the lower removal rates may be related to the mass loss of OS-M(P) during regeneration. Figure 8d shows the mass changes of OS-M(P) and OS-M(G) after each adsorption–desorption. The mass loss of OS-M(P) during regeneration is large. The recovery rate of OS-M(P) after 3 regenerations is 20%, while the recovery rate of OS-M(G) after 5 regenerations is 75.75%. Therefore, OS-M(G) has a higher practical value than OS-M(P) due to its better reusability and higher recovery rate. In order to explore the adsorption potential of OS-M(P) and OS-M(G) for actual AMD, adsorption experiments were carried out. The actual AMD was collected from the mine water of a lead–zinc mine in Huludao City, Liaoning Province, China. The initial concentrations of typical heavy metal ions (Pb, Cd, Fe, Mn, Cu, and Zn) and anions (SO_4_^2−^) present in actual AMD and the adsorption concentrations of OS-M(P) and OS-M(G) were determined, as shown in Table 7. The removal rates of OS-M(P) and OS-M(G) for actual AMD are shown in Fig. 8e. It was found that even though there was competitive adsorption between heavy metal ions, the removal rates of heavy metal ions and sulfates of the two adsorbents were higher, and the overall treatment effect of OS-M(G) was better than that of OS-M(P).

**Table 7:** The concentration of heavy metal ions and anions before and after adsorption in actual AMD.

Actual AMD	Heavy metal ions						Anions
	Pb(II)	Cd(II)	Fe(II)	Mn(II)	Cu(II)	Zn(II)	SO_4_^2−^
Initial concentrations (mg/L)	48.25	13.32	3.14	39.31	8.2	77.55	3479
Concentrations after adsorption (mg/L)							
OS-M(P)	5.96	1.19	0.18	8.09	1.45	8.96	998
OS-M(G)	4.13	0.88	0.11	6.49	0.93	8.19	934

## Conclusion

Two adsorbents, OS-M(P) and OS-M(G), in powdery and globular morphologies, were successfully prepared from lignite and oyster shells for the removal of Pb(II) and Cd(II) from AMD. They play an important role in the adsorption and polymerization of Pb(II) and Cd(II) through electrostatic adsorption, neutralization and precipitation, ion exchange and complexation. However, the adsorption properties of the two adsorbents are different. The adsorption process of OS-M(P) follows the pseudo-second-order kinetic model and Langmuir model, and the maximum adsorption saturation capacity of Pb(II) and Cd(II) are 332.6219 mg/g and 318.9854 mg/g (25 °C), respectively. The adsorption process of OS-M(G) conforms to the pseudo-second-order kinetic model and Freundlich model, the maximum adsorption saturation capacity of Pb(II) and Cd(II) are 93.6463 mg/g and 6.7822 mg/g (25 °C), respectively. The saturated adsorbent can slowly desorb Pb(II) and Cd(II) at different pH levels, in particular, the desorption effect is best when pH is 11, and the desorption rates of OS-M(G) for Pb(II) and Cd(II) are 74.26% and 63.57%, respectively. while OS-M(G) has obvious advantages over OS-M(P) in terms of regeneration times (5 times), adsorbent recovery rate (75.75%) and treatment capacity of actual AMD wastewater. Therefore, OS-M(G) has the potential for large-scale applications and can be used as a promising environmentally friendly adsorbent for the long-term repair of AMD.

## Data Availability

This manuscript includes all the generated or analysed data during the present study.
